# Towards Inertial Sensor Based Mobile Gait Analysis: Event-Detection and Spatio-Temporal Parameters

**DOI:** 10.3390/s19010038

**Published:** 2018-12-22

**Authors:** Wolfgang Teufl, Michael Lorenz, Markus Miezal, Bertram Taetz, Michael Fröhlich, Gabriele Bleser

**Affiliations:** 1 Junior Research Group wearHEALTH, Technische Universität Kaiserslautern, Gottlieb-Daimler-Str. 48, 67663 Kaiserslautern, Germany; lorenz@cs.uni-kl.de (M.L.); miezal@cs.uni-kl.de (M.M.); taetz@informatik.uni-kl.de (B.T.); bleser@informatik.uni-kl.de (G.B.); 2 Department of Sports Science, Technische Universität Kaiserslautern, Erwin-Schrödinger-Str. 57, 67663 Kaiserslautern, Germany; michael.froehlich@sowi.uni-kl.de

**Keywords:** accelerometer, gyroscope, inertial measurement unit, ambulatory motion analysis, gait parameters, wearable sensors

## Abstract

The aim of this study was to assess the validity and test-retest reliability of an inertial measurement unit (IMU) system for gait analysis. Twenty-four healthy subjects conducted a 6-min walking test and were instrumented with seven IMUs and retroreflective markers. A kinematic approach was used to estimate the initial and terminal contact events in real-time. Based on these events twelve spatio-temporal parameters (STP) were calculated. A marker based optical motion capture (OMC) system provided the reference. Event-detection rate was about 99%. Detection offset was below 0.017 s. Relative root mean square error (RMSE) ranged from 0.90% to 4.40% for most parameters. However, the parameters that require spatial information of both feet showed higher errors. Step length showed a relative RMSE of 6.69%. Step width and swing width revealed the highest relative RMSE (34.34% and 35.20%). Test-retest results ranged from 0.67 to 0.92, except for the step width (0.25). Summarizing, it appears that the parameters describing the lateral distance between the feet need further improvement. However, the results of the validity and reliability of the IMU system encourage its validation in clinical settings as well as further research.

## 1. Introduction

Gait analysis is an important tool in the evaluation of operative procedures [1,2], rehabilitation progress [3], or the assessment of the motor status in neurologically impaired patients [4,5]. There are various parameters that are of interest such as joint kinematics (JK), spatio-temporal parameters (STP), joint forces, pressure distributions, and muscle activities. There are also various systems for the measurement/estimation of the above mentioned variables. However, most systems are specialized on the measurement of a subset of these parameters. Few systems can cover a wide range of parameters. For the measurement of 3D JK and STP it is common to use a marker based optical motion capture (OMC) system in combination with force or pressure plates [6,7]. However, OMC systems tend to be expensive, laboratory-bound and their usage tends to be time consuming and needs expert knowledge. The introduction of inertial measurement units (IMU) and magnetic inertial measurement units (MIMU) in the motion analysis [8,9] presented the research community with a wide range of possibilities in the assessment of gait.

IMU/MIMU systems are used to calculate 3D JK in different settings [10,11,12] and IMU/MIMU derived STP are increasingly discussed in recent literature [13,14]. Caldas et al. [14] reported that event-detection, initial contact (IC) and terminal contact (TC), and its robustness and delay are important factors when calculating STP, especially in real-time applications. They further state that an IC accuracy of only 90% could be achieved when using only a gyroscope. However, detection accuracy was improved when additionally incorporating accelerometer data. Seel et al. [15] and Müller et al. [16] introduced an online gait phase detection algorithm with automatic adaptation to gait velocity changes based on one foot/shoe-mounted IMU. The mounting orientation on the foot is arbitrary. They also tested their algorithm with healthy people as well as with transfemoral amputees with a leg prosthesis and stroke patients.

There are already numerous IMU/MIMU systems based on one or two sensors [4,15,16,17,18]. However, these systems deliver mostly STP that incorporate the spatial information of one foot, stride length, stride time, stance time, or swing time. Parameters that depend on the spatial relation of both feet, e.g., step length and step width, are more complex to calculate and therefore there is a paucity of literature on this problem [18,19,20,21].

Takeda et al. [20] developed the IMU based system “H-Gait”. A kinematic approach based on seven IMUs which is able to deliver 3D JK and STP including step length and step width. However, in the validation of “H-Gait” [22] only sagittal angles and temporal parameters are mentioned. Köse et al. [19] used a single IMU attached to the sacrum to calculate left and right step length. However, this approach was specialized on the estimation of step length and step duration only. Cimolin et al. [23] also installed a single IMU on the lower back of 18 normal and obese subjects for the estimation of stride and step length. The step length was calculated based on an inverted pendulum model. However, Cimolin et al. [23] did not report results for the step length. Bertuletti et al. [21] developed a device consisting of an infrared time of flight proximity sensor and an MIMU to measure the distance between the feet at swing-through. This approach was validated with a mechanical pendulum and a small sample of one human subject. However, this apparatus needs a rather complex set-up procedure.

In general, there are few validated systems that deliver 3D joint kinematics and STP [22,23,24,25]. One commercial MIMU system, consisting of two or seven MIMUs offers the possibility of measuring a collection of STP as well as the sagittal joint angles of hip, knee, and ankle. This system was validated by Nüesch et al. [24] and Donath et al. [25,26].

The intention of the authors is the development of an IMU system for complex 3D gait analysis, delivering full 3D JK of the lower limbs and pelvis and a wide range of STP including parameters that depend on the spatial information of one as well as both feet. In addition, the present system features real-time event-detection and kinematic estimation, which is relevant in different applications; e.g., it provides the possibility of online feedback coupled to specific gait events/phases and related kinematic parameters for supporting gait interventions in clinical settings, see [27] for a review. Shull et al. [28] provided real-time vibrotactile feedback on multiple kinematic parameters (foot progression angle, tibia angle, trunk sway) measured through an OMC system to support gait retraining for knee osteoarthritis patients. In [29], vibrotactile feedback was coupled to gait events measured through insoles for supporting stroke patients in improving their gait symmetry. Crea et al. [30] restored the somatosensory feedback of special gait events in transfemoral amputees via vibrating elements on the thigh. These were controlled through the detection of gait events via pressure insoles. Another application concerns the real-time control of neuroprostheses as proposed, e.g., in Seel et al. [31] and Valtin et al. [32]. They developed a tool for foot eversion/inversion control and selective muscle activation in patients with drop foot. Their approach was based on the IMU derived kinematics of the foot. All of these applications require accurate and real-time estimation/detection of kinematic parameters and/or gait events.

A validation of the 3D JK calculated based on an initial version of the system used in this study was recently published [33]. Therefore, it is the continuative aim of this study to validate the accuracy of IMU based event-detection and STP, both calculated using a kinematic model approach.

## 2. Materials and Methods

### 2.1. Subjects and Data Acquisition

**Figure 1:**
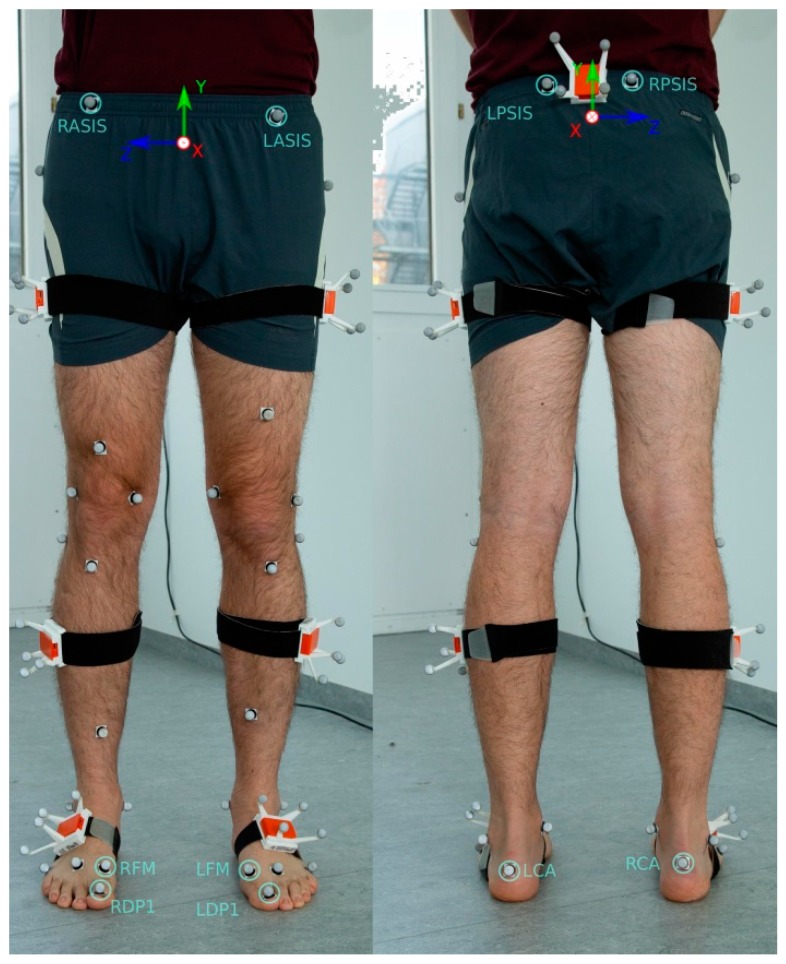
Model picture of the instrumentation with the retroreflective markers and inertial measurement units (IMU). The IMUs were inserted into matched rigid boxes for additional analysis not relevant in the present examination. The markers used for the identification of the initial contact (IC) and terminal contact (TC) events are marked. The calcaneus (CA) markers were used to identify IC, the first distal phalanx (DP1) markers were used to identify the TC in the optical system. In the IMU system virtual representations of the CA and first metatarsal (FM) markers were used to identify the events (see Figure 3). Right Anterior Spina Iliaca Superior (RASIS), Left Anterior Spina Iliaca Superior (LASIS), Right Posterior Spina Iliaca Superior (RPSIS) and Left Posterior Spina Iliaca Superior (LPSIS) markers were used to define the pelvic coordinate system. Consider that in the actual study the above mentioned relevant markers were attached directly onto the skin rather than on clothing.

24 healthy subjects were included in the study (12 female, 12 male). The study was approved by the ethical committee of the Technische Universität Kaiserslautern (TUK) and meets the criteria of the declaration of Helsinki. After receiving all relevant study information, the participants signed an informed consent to the study including a permission to publish data. Each participant conducted two test sessions on two different days with approximately seven days in between. The subjects were instrumented by means of seven IMUs (MTW Awinda, Xsens Technologies BV, Enschede, The Netherlands) attached to the segments of the lower body and pelvis. The pelvis IMU was attached to the segment roughly at half the distance between the left and right spinae illiacae posteriores superiores. Each thigh IMU was attached to the segment approximately at half the distance between the greater trochanter and the lateral epicondyle. Each shank IMU was attached roughly at half the distance between the lateral epicondyle and the lateral malleolus. Each foot IMU was attached roughly at three-fourth the distance between the calcaneus and the head of the second metartarsal. Further, 32 retroreflective markers were attached to bony landmarks according to Leardini et al. [34] (Figure 1). In the present study only the markers attached to the calcaneus (CA), the first distal phalanx (DP1) and the four markers creating the pelvic segment were considered.

**Figure 2:**
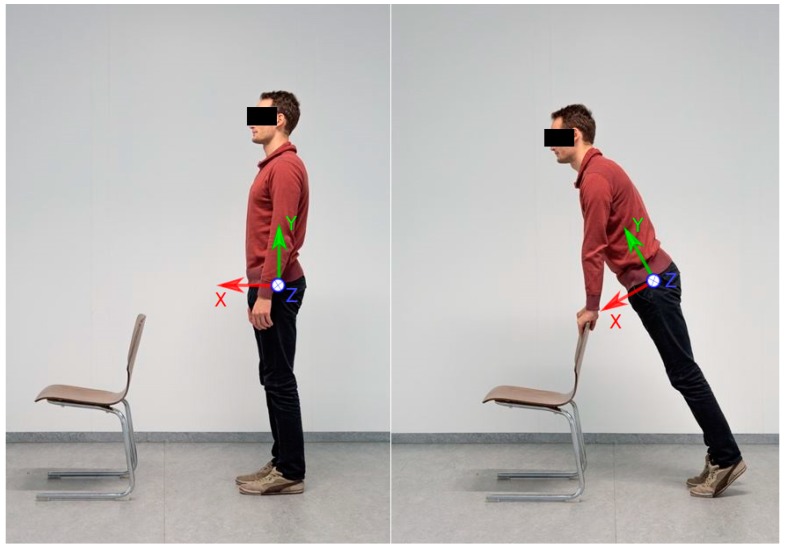
Demonstration of the two-step-calibration process. On the left side the subject is standing in neutral zero position. On the right side, the subject is slightly inclined forward, so that every lower body segment is rotated only around the z-axis shown in the figure (frontal body axis).

A test session consisted of one 6-min walk test. Prior to the main test a variation of the two-step-calibration poses described by Palermo et al. [35] was conducted. The participants had to maintain a slightly inclined standing position for several seconds and then stand in a neutral zero position for another several seconds (Figure 2). The underlying assumptions are: in the neutral zero pose, all segments are aligned with gravity, the feet are parallel and pointing forward in the sagittal plane, i.e., they are neither outward nor inward rotated. Moreover, it is assumed that between the two poses every segment and IMU is rotated around the frontal body axis only, while the amounts of rotation can differ between segments. Subjects then were asked to walk along a straight line of about 5 m. On both ends of the line, the subjects had about 1 m additional space to turn sharply and then walk the line straight back again. The areas including the turning phases were omitted for the evaluation.

IMU and OMC data were hardware-synchronized, using a standard 5V transistor-transistor-logic signal, and recorded at 60 Hz using Xsens MVN Biomech (Version 4.3.7, Xsens Technologies BV, Enschede, The Netherlands) and OptiTrack Motive (Version 1.10.0, NaturalPoint, Inc., Corvallis, OR, USA).

**Figure 3:**
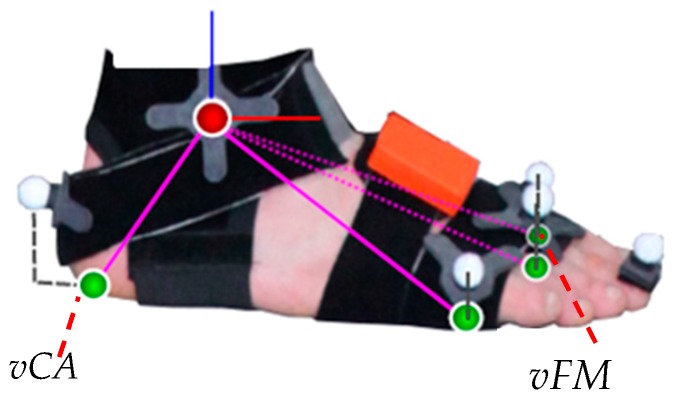
Foot model with optical markers and the four projected virtual contact points (green spheres) used for both the six degrees of freedom segment kinematics estimation and the IMU based gait event-detection. The virtual CA (vCA) marker and the virtual FM (vFM) marker, which were used for the event-detection in the IMU system, are denoted. The figure has been taken from [37].

The sensor-fusion method for obtaining the 6 degrees of freedom segment kinematics from the IMU data is summarized in [33] and based on [36,37]. Note, the segment kinematics serve as basis for the gait event-detection as well as for calculating the STP. The magnetometer-free kinematics estimation method fuses gyroscope and accelerometer measurements with assumptions from a per-segment motion model, biomechanical model constraints and environmental constraints in an iterated extended Kalman filter framework. For this, the state contains IMU-centered kinematics (global position, velocity, acceleration, orientation, angular velocity) of all seven lower body segments, which are all jointly estimated. These are related to the segment kinematics via the IMU-to-segment calibrations, which are assumed known and rigid. Note, the resulting coupled estimation of the complete lower body movement was already shown to provide drift-free joint kinematics estimates even without using magnetometer information in [33]. The biomechanical constraints model the fact that the body segments are connected at the joints. For this, the segment lengths are assumed known. The joints are all modeled with 3 degrees of freedom. The environmental constraints consist of estimated ground contacts at virtual foot contact points suggesting zero height and zero velocity pseudo measurements, since a level ground is currently assumed (Figure 3). The probabilistic ground contact estimation method is carried out in parallel to the kinematics estimation for a set of potential ground contact points as shown in Figure 3, without making any assumptions concerning the type of movement (see [37] for more details). Hence, the segment kinematics estimation method as well as the calculation of the spatio-temporal parameters given the events are generally applicable, while only the chosen event-detection method is specific for gait. Compared to the description in [33], only the noise settings of the connected segments, the zero height and the zero velocity pseudo measurements were changed ($\Sigma^{p}=10^{-8}\times I_{3x3},\;\Sigma^{\dot{p}}=10^{-4}\times I_{3x3},\;\Sigma^{z}=5\times 10^{6}$). Note that the virtual contact points of the IMU system represent the marker positions in the OMC system projected on the foot soles [37] (Figure 1). The DP1 marker was omitted for the foot model in the IMU system, since no inertial information is available to estimate the orientations of the proximal and distal phalanges.

In [33], the biomechanical model (including the segment lengths), the IMU-to-segment calibrations and the tracking initialization were extracted from the OMC data. In the present study, the biomechanical model was obtained from the Twente Lower Extremity Model (TLEM) 2.0 dataset [38] which was scaled based on the subject’s gender and height using anthropometric tables [39]. The IMU-to-segment orientations were obtained from the above mentioned calibration poses according to [35] and the tracking process was initialized from inertial data only by assuming a neutral zero position. The IMU-to-segment positions were assumed fixed in the kinematic model. The assumed positions along the segments coincide with the description of the actual IMU positioning at the beginning of Section 2.1. The distances out of the segments were all assumed with 3 cm to roughly account for the tissue lying between bones and sensor origins. More specifically, the pelvis IMU position was assumed at 3 cm along the negative x-axis (see Figure 1), the IMUs attached to thighs and shanks were assumed at 3 cm along the positive (left) or negative (right) y-axis (pointing laterally, see [33]), and the IMUs attached to the feet were assumed at 3 cm along the positive z-axis (see Figure 3).

The real-time detection of IC and TC events, as mentioned above, uses a kinematics based algorithm. It is a modification of the coordinate-based treadmill algorithm described in Zeni et al. [40] for over-ground walking. The general idea of the base algorithm is that the IC corresponds to the heel having maximal distance to the pelvis center in walking direction, while the TC corresponds to the toe having maximal distance to the pelvis center against the walking direction. In the present study, this algorithm was applied to both the OMC data (optical markers on pelvis and feet) and the IMU data (estimated pelvis pose and virtual foot markers in the biomechanical model). Note, an offline version was used for deriving the events from the OMC data, while an online version was developed for obtaining real-time event-detections. The required modifications (compared to [40]) for over-ground walking and real-time detection are described in the following.

First of all, the walking direction, which changed with each turn in the present study (in contrast to the treadmill walking in [40]), was extracted from the captured data by filtering the estimated x-axis of the pelvis segment. The latter is a three dimensional unit vector lying in the sagittal plane and pointing in anterior direction for both the biomechanical model used to process the OMC and the IMU data (see Figure 1). The filtering was applied to compensate for small internal and external rotations which naturally appear around the direction of movement during walking. It was based on an autoregressive model of first order (AR 1). After each filtering operation the axis was renormalized to unit length. In the offline version (for extracting the events from the OMC data), the filter was used in a zero-lag manner to avoid the introduction of a delay.

**Figure 4:**
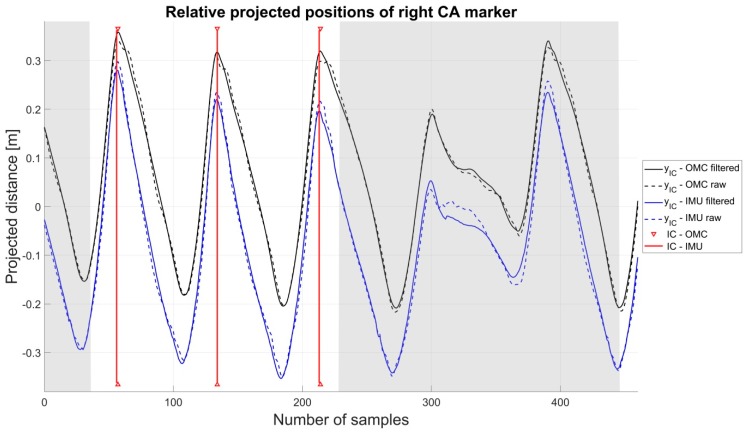
The shadowed areas indicate the turning phases. The offset between the OMC and IMU system originates in the different positions of the virtual and real heel marker as well as the different positions of the pelvis center.

To obtain the IC and TC events, the relative positions of the respective real or virtual foot markers with respect to the sacrum were computed. These distances were then projected onto the estimated walking direction using the dot product. As in the original algorithm, the gait events were then extracted by finding the peaks of the projected distances. The maxima in the projected distances $y_{IC}$ between the CA marker positions and the pelvis center positions defined the ICs and the minima in the projected distances $y_{TC}$ between the DP1 marker positions (for the OMC data) or the FM marker positions (for the IMU data) and the pelvis center positions defined the TCs. An exemplary course of the projected distances $y_{IC}$ for both systems with filtered and unfiltered x-axis is illustrated in Figure 4.

In the offline version, the Matlab function findpeaks was used and the median of the complete sequence was used as a threshold for the minimal peak height.

In the online version, the extrema were detected as zero-crossings of differences of subsequent values of the projected distances $y_{event}$ with *event* ∈{IC,TC}. Instead of using the median as threshold for peak detection, the online version validates each found peak using the following conditions:

1. At the found peak, $y_{IC}>0$ or $y_{TC}<0$, i.e., the IC occurs in anterior and the TC in posterior direction.

2. After an event has been detected, a subsequent event of the same type cannot occur within a given amount of time (0.42 s in our experiments).

3. In addition to the previous temporal condition and in order to increase the robustness of the algorithm to false positive detections, a spatial condition based on the distance between two subsequent events has to be fulfilled. The absolute value of the projected distances between the current event and the last event $\left|y_{event}-y_{lastevent}\right|$ can be interpreted as the distance between the marker positions associated to IC and TC or vice versa with respect to the pelvis segment center. We assume this distance to be $|y_{event}-y_{lastevent}|>\;h_{min,event}$, where $h_{min,event}$ indicates a threshold.

For the update of the threshold, we again use a filter based on a AR 1 model to compensate for greater fluctuations of the distances over time. After a valid event-detection the threshold is updated according to $h_{min,event}=\left(3\;h_{previous,min,event}+1\;{\bar{h}}_{min,event}\;\right)/4$. Here $h_{previous,min,event}$ denotes the threshold of the previous event and the value ${\bar{h}}_{min,event}$ is computed as ${\bar{h}}_{min,event}\;=\;a\;\left|y_{event}-y_{lastevent}\right|\;$. To ensure that also smaller strides are detected after several bigger strides, the scaling factor a∈]0, 1] was introduced. In our setting it was chosen to be 0.7. The threshold was initialized with 1.2 times the foot size. Note, these parameters were empirically determined during pilot experiments and then used for the present study. The pilot experiments showed that the detection is not sensitive to the exact choice of the parameters.

Based on the detected events and estimated real/virtual foot marker positions, the STP parameters described in Table 1 were calculated and the combined average of both lower limbs was considered for evaluation.

### 2.2. Statistical Analysis

To evaluate the performance of the event-detection algorithm all erroneous detections of the IMU system were captured (surplus and missing events compared to the OMC system) and the relative error compared to the OMC system was calculated. Further, the average detection offset (time difference between events in the IMU and OMC system) for all subjects was calculated for IC and TC. Therefore, the detection offsets over all subjects were averaged and converted from frames per second (fps) to seconds according to the data acquisition frame rate of 60 Hz. Additionally, the detection offset of IC and TC was graphically represented.

To validate the results of the STP the mean error, the root mean square error (RMSE) plus 95% confidence interval (CI), the relative RMSE and Bland-Altman (BA) analysis were calculated. In the present evaluation the left and right side were combined.

The chi-square goodness-of-fit test was used to check for normal distribution in the data. Consequently, a paired sample t-test or the non-parametric Wilcoxon rank sum test was calculated to find significant differences in the STP between the IMU and OMC system. The critical *p*-value was set to α = 0.05.

To evaluate the test-retest reliability of the STP the intraclass correlation coefficient (ICC) was estimated. In this study a two way random effect model was used to calculate the reliability of the average of two measurements according to McGraw and Wong [41]. ICC values below 0.40 were rated poor, values between 0.41 and 0.59 fair, between 0.60 and 0.74 good and above 0.74 excellent [42].

All statistics, the calculation of the events from the OMC data and the calculation of the STP were conducted in Matlab 2017 (Mathworks Inc., Natick, MA, USA). The real-time event-detection from IMU based kinematics data was implemented in C++.

## 3. Results

### 3.1. Validity

In the following, the results of the left and right lower leg were combined for the evaluation. Differences between sides were below 0.006 m in the spatial parameters and below 0.004 s in the temporal parameters. The difference in the cadence of left and right side was 0.48 steps/min.

**Figure 5:**
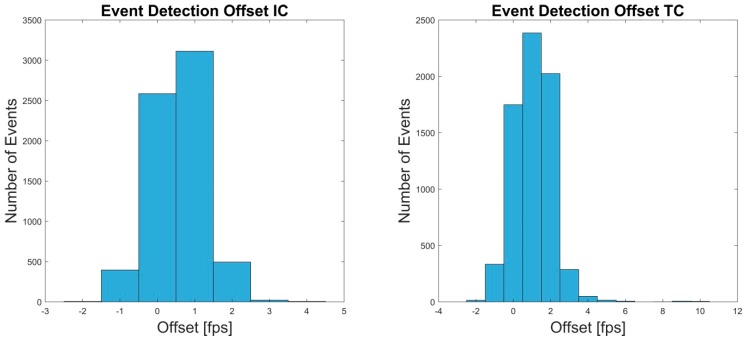
The Offset in frames per second (fps) between OMC and IMU system for the IC and TC events of test 1 are shown.

A total of 13,415 events were detected by the OMC system, IC, and TC combined. The detection error of the IMU system was below 1.2%. The detection offset was below 0.017 s. A detailed description of the results can be found in Table 2 and Figure 5.

Mean OMC and IMU values of the different STP as well as the p-values of the significance test, the mean error, the RMSE ± SD and 95% CI, relative RMSE and BA bias are shown in Table 3. Low errors and high agreement were observed over all parameters. Relative errors were below 7% with exception of the step width and swing width. The step width displayed a relative RMSE of 34.34%. The swing width revealed a relative RMSE of 35.20%. These values correspond to a RMSE of 0.03 m. The average step width as well as swing width measured at 0.09 m in the OMC and 0.10 m and 0.08 m in the IMU system, respectively. Further, the STP dependent on the spatial information of left and right CA or DP1/FM marker showed higher relative RMSE compared to the remaining parameters.

**Figure 6:**
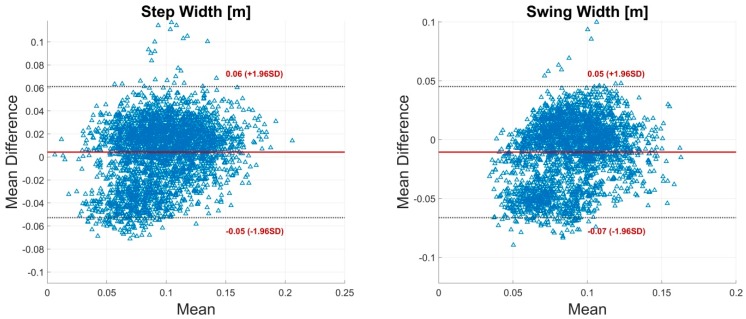
Bland-Altman (BA) plots for step width and swing width. Each plot contains all calculated data points of one parameter of all subjects. The solid line indicates the mean difference. The dashed lines indicate the limits of agreement (LoA) (95% CI of the mean difference).

**Figure A1:**
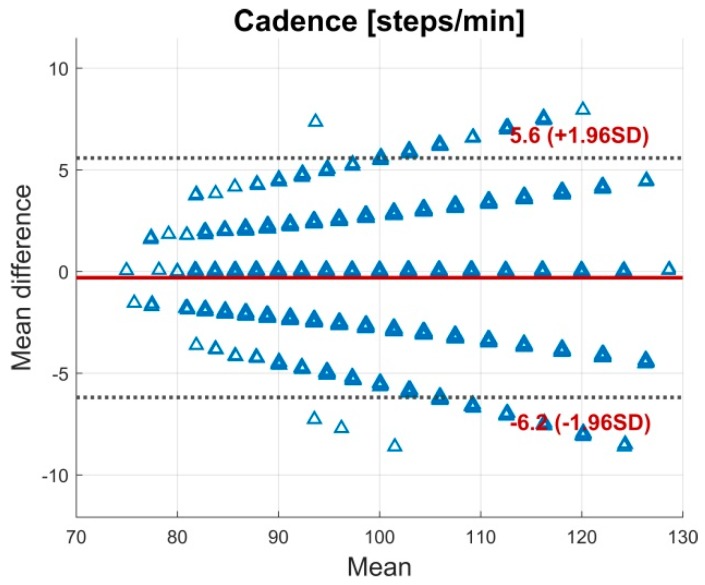
BA plot for the cadence. The plot contains all calculated data points of all subjects. The solid line indicates the mean difference. The dashed lines indicate the LoA (95% CI of the mean difference).

**Figure A2:**
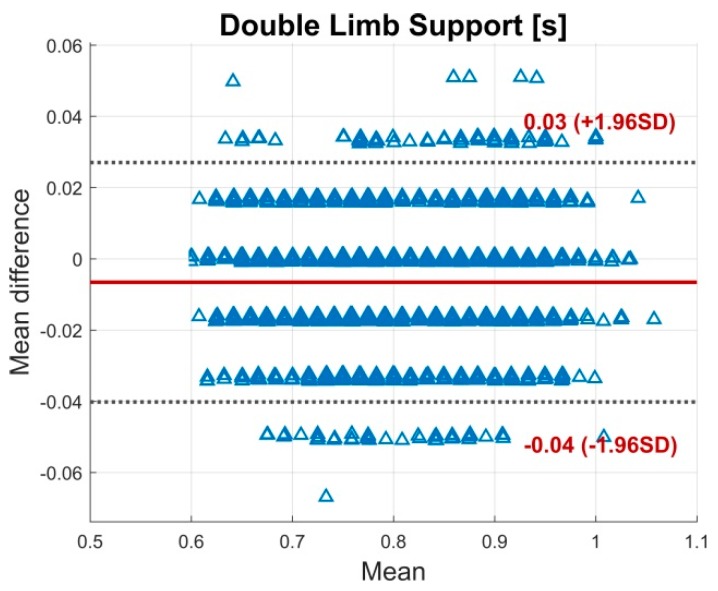
BA plot for the double limb support. The plot contains all calculated data points of all subjects. The solid line indicates the mean difference. The dashed lines indicate the LoA (95% CI of the mean difference).

**Figure A3:**
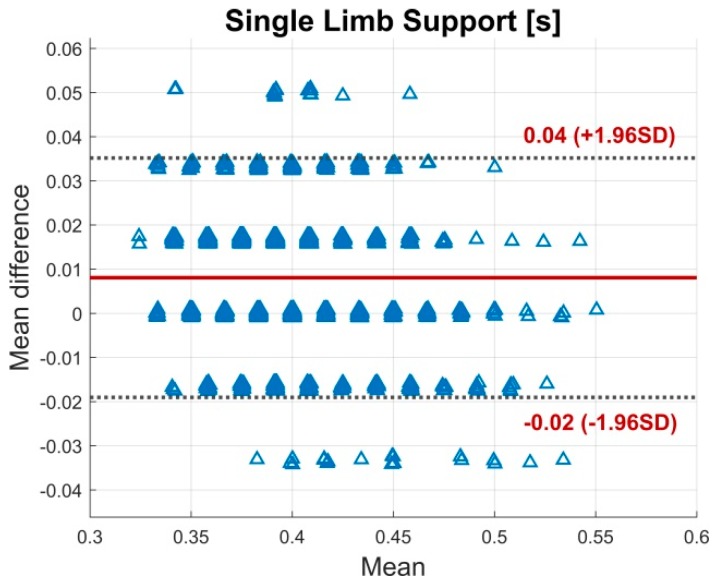
BA plot for the single limb support. The plot contains all calculated data points of all subjects. The solid line indicates the mean difference. The dashed lines indicate the LoA (95% CI of the mean difference).

**Figure A4:**
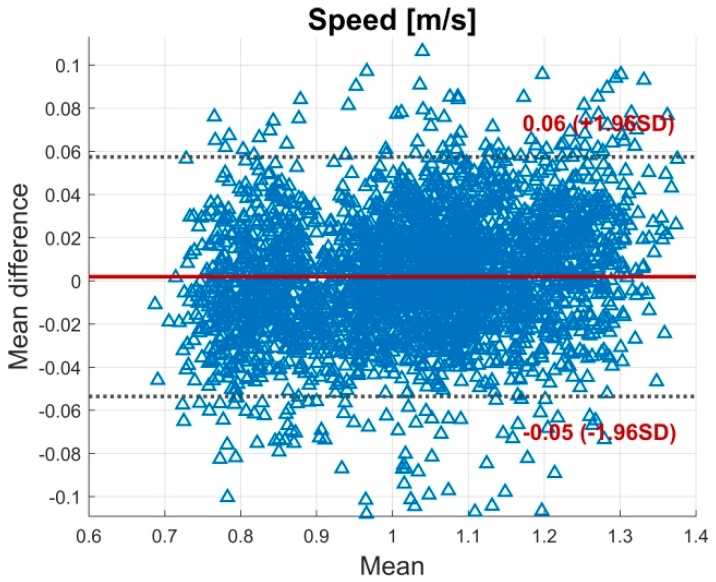
BA plot for the speed. The plot contains all calculated data points of all subjects. The solid line indicates the mean difference. The dashed lines indicate the LoA (95% CI of the mean difference).

**Figure A5:**
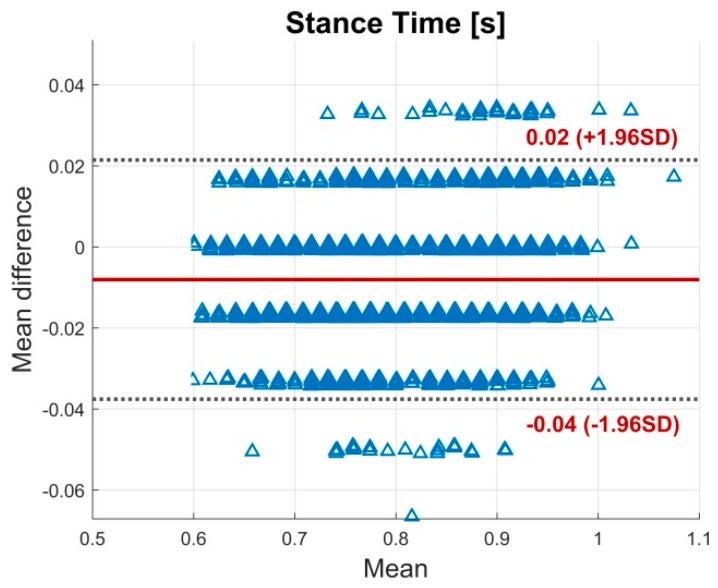
BA plot for the stance time. The plot contains all calculated data points of all subjects. The solid line indicates the mean difference. The dashed lines indicate the LoA (95% CI of the mean difference).

**Figure A6:**
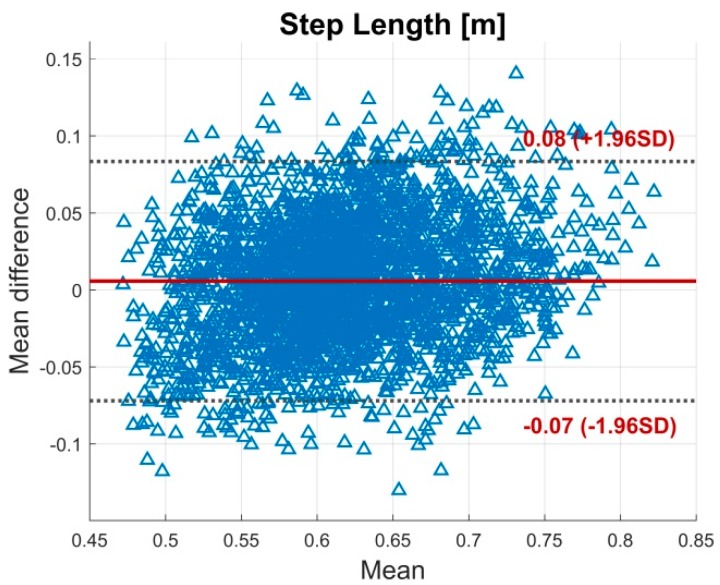
BA plot for the step length. The plot contains all calculated data points of all subjects. The solid line indicates the mean difference. The dashed lines indicate the LoA (95% CI of the mean difference).

**Figure A7:**
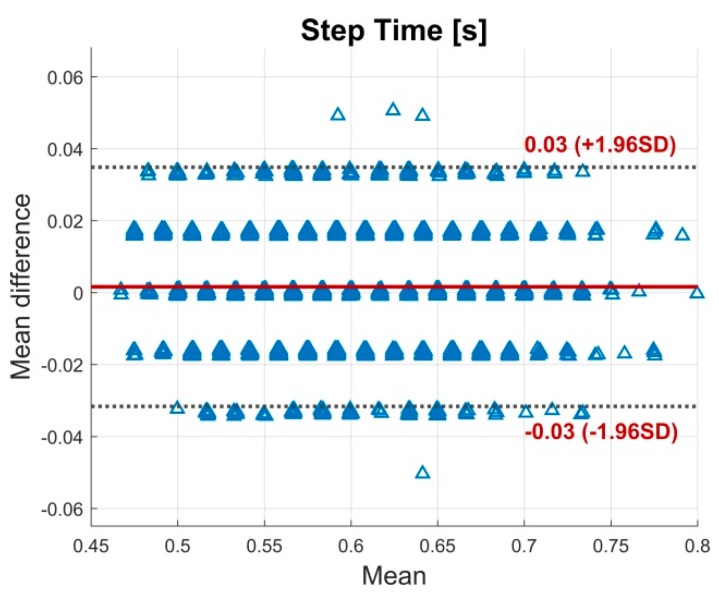
BA plot for the step time. The plot contains all calculated data points of all subjects. The solid line indicates the mean difference. The dashed lines indicate the LoA (95% CI of the mean difference).

**Figure A8:**
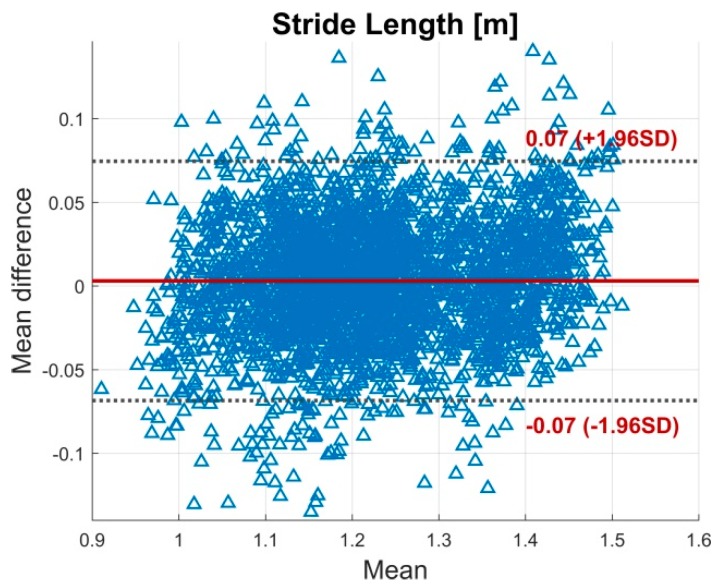
BA plot for the stride length. The plot contains all calculated data points of all subjects. The solid line indicates the mean difference. The dashed lines indicate the LoA (95% CI of the mean difference).

**Figure A9:**
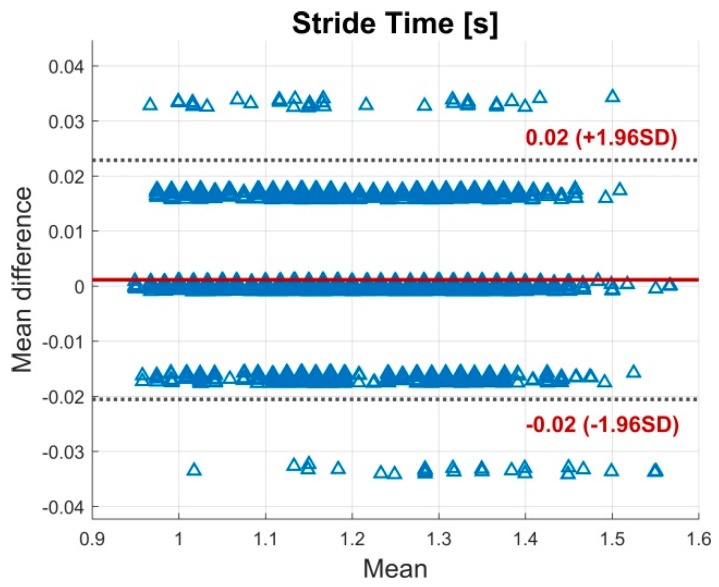
BA plot for the stride time. The plot contains all calculated data points of all subjects. The solid line indicates the mean difference. The dashed lines indicate the LoA (95% CI of the mean difference).

**Figure A10:**
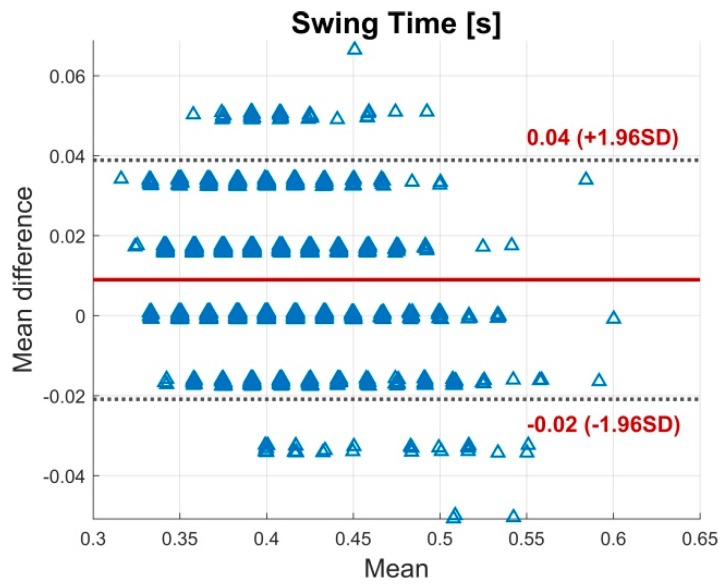
BA plot for the swing time. The plot contains all calculated data points of all subjects. The solid line indicates the mean difference. The dashed lines indicate the LoA (95% CI of the mean difference).

The BA analysis showed high agreement between the IMU system and the reference system for most of the parameters. Step width and swing width showed low biases but rather wide limits compared to the dimension of the actual parameter value. Figure 6 shows exemplary BA diagrams for step width and swing width. Additional BA diagrams (Figure A1, Figure A2, Figure A3, Figure A4, Figure A5, Figure A6, Figure A7, Figure A8, Figure A9 and Figure A10) can be found in Appendix A.

### 3.2. Test-Retest Reliability

**Figure 7:**
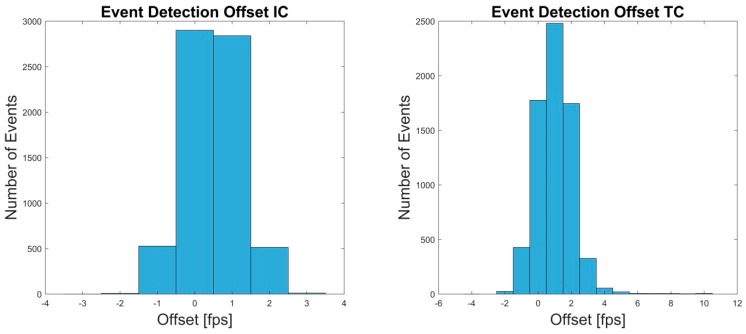
The Offset in frames per second (fps) between OMC and IMU system for the events IC and TC of retest are shown.

The event-detection of the retest revealed slightly more erroneous detections in both IC and TC. However, percentage error was below 1.60%. Detection offsets did not differ from Test 1. For details see Table 4 and Figure 7.

The ICC revealed excellent test-retest correlations for all parameters except swing time, step width, swing width and step length. Step width was rated poor (0.25). Step length, swing width, and swing time were rated good (0.67, 0.69, and 0.73). The ICC calculation for the OMC system showed good to excellent values over all parameters (>0.67). All ICC values for the IMU and OMC system are shown in Table 5.

## 4. Discussion

### 4.1. Validity

The present evaluation of events and STP obtained from a real-time IMU based kinematic model approach revealed high validity compared to the reference system. The event-detection algorithm used in this study was designed for a kinematic approach in overground and treadmill walking. The algorithm was validated in Zeni et al. [40]. Therefore, it was considered a valid approach for the detection of IC and TC and was employed in this study rather than an alternative approach [43].

The detection rate of the event-detection was about 99% for IC and TC. Further, the detection offset was below 0.010 s for IC and below 0.017 s for TC. Storm et al. [44] found a similar detection offset for IC but a higher error for TC (0.051 s). Bertoli et al. [4] found a detection offset of −0.009 s to 0.009 s for IC and TC. However, their detection approach partly requires offline calculations.

Müller et al. [16] found a mean delay for TC of approximately 0.1 s and approximately 0.05 s for IC. However, consider that their recording frequency was restricted to 50 Hz. Further, they used a different algorithm for the event-detection in the reference system. However, Müller et al. [16] and Seel et al. [15] showed that their system does not depend on the knowledge of an accurate IMU–to-segment calibration, since mostly accelerometer and gyroscope measurement norms are considered for the detection of the gait phase transitions. Moreover, the rotation of the global coordinate system, which is required for obtaining the IMU velocity through integration of the acceleration measurements as basis for toe off detection, is dynamically determined as the rotation of the IMU at foot rest.

A limitation of the study was that the IMU based kinematic model did not provide a virtual marker on DP1. Therefore, the virtual marker FM was used for TC detection. This might have led to an increased detection offset between OMC and IMU system. Consider, the position of the CA marker of the IMU system was projected on the sole whereas the CA marker of the OMC system was located on the dorsal aspect of the calcaneus (Figure 3). Another drawback of the study was that the IMU-to-segment positions were assumed fixed rather than, e.g., estimated from IMU data. Therefore, differences between the assumed IMU positions in the model and the true positions on the segments could have appeared. The effect of these deviations on the estimated IMU orientations was examined for slow and fast movements in [36]. For example, a simulation study in [36] showed that a deviation of about 10 cm along the segment could lead to a mean angular error of up to approximately 5° in fast movements when not using magnetometer information (up to about 6.5° for 10 cm out of segment deviations). However, there is work in progress to develop pose-independent and movement-independent calibration methods estimating not only the IMU-to-segment orientations but also the IMU-to-segment positions [45,46].

The temporal parameters and those that are dependent on the spatial information of one foot showed the lowest relative RMSE. Stride time revealed the lowest relative error, 0.90% (RMSE 0.01 s), for all parameters. These results are comparable to Kluge et al. [18] who found similar results for stride time. They examined a commercial IMU system consisting of two IMUs attached to the lateral aspect of the heels. Their results were compared to a marker-less OMC system. They further presented results for stance time (5.40%, RMSE 0.04 s), swing time (8.20%, RMSE 0.04 s), stride length (3.60%, RMSE 0.05 m), and speed (3.60%, RMSE 0.04 m/s). For all these parameters the present system revealed better results. Consider that the subjects in the study of Kluge et al. [18] displayed a higher overall stride length (~1.45 m) and a slightly lower stride time (~1.13 s).

Bertoli et al. [4] investigated a large sample of subjects (236) across different clinics and states of impairment. They used two MIMUs attached above both ankles and a pressure mat as a reference system. Further, they used an algorithm, assumed to be partially offline, based on the identification of trusted swing and stance phases of the lower limbs for the event-detection according to Trojaniello et al. [47]. They found rather good results concerning the validity of the temporal parameters and the stride length. The mean error of the stride length measured from −0.001 m to −0.014 m for the healthy group across the different clinics. Stride time, swing time, step time and stance time were all below 0.025 s. Therefore, they present similar results concerning the temporal parameters compared to the present study.

As addressed in the introduction Donath et al. [25] validated the STP of a commercial MIMU system combining the measurement of 1D (sagittal) JK and STP. They calculated RMSE for stride length (0.06 m), stride time (0.04 s), speed (0.08 m/s) and cadence (0.09 steps/min). The present system revealed better results concerning the stride length (0.04 m), stride time (0.01 s) and speed (0.03 m/s). However, the cadence showed a distinctively higher RMSE (3.10 steps/min). It is not clear how the cadence was estimated in the system used by Donath et al. [25]. In the present study, the cadence was calculated incorporating the step time (see Table 1).

As mentioned above there are few studies that measured IMU based spatial parameters dependent on the position information of both feet. Cimolin et al. [23] used one IMU attached to the pelvis to measure STP. They delivered a theoretical approach for the measurement of the step length, based on leg length and an inverted pendulum gait model. However, they omitted the results of the calculation.

Köse et al. [19] used a similar setting and came up with a special approach for the calculation of the step length. They assumed that the pelvic displacement along the line of progression between an ipsilateral IC and a contralateral IC equals the step length. They found promising results with a mean error of 0.009 m compared to an OMC system. The present study revealed a mean difference of 0.006 m. However, Köse et al. [19] examined a small sample size of 9 subjects and used a partial-offline calculation.

It was a special challenge of the present work to calculate the parameter step width. The step width was described as the most sensitive parameter for gait categorization in older adults [48]. Further, step width is an important evaluation parameter concerning cerebellar ataxic gait [49]. Ilg et al. [50] found a significant correlation of step width with the International Cooperative Ataxia Rating Scale. Stolze et al. [49] found that step width altered about 7 cm between a healthy and a cerebellar impaired population. The BA analysis of the present study revealed a bias of ±0.06 m regarding the step width (Figure 6). These results indicate that the differentiation between healthy and impaired subjects would be challenging with the current system. Thus, the measurement of the step width has to be further improved. It might have been that imprecisions in the segment length scaling and the neutral zero position led to increased errors in the step width. The integration of an individualized biomechanical model and a pose-independent calibration might improve these results.

To the knowledge of the authors, there are no studies that report step width measurement based on IMUs that use the same definition of the parameter as presented in this work. Müller et al. [51] tried to measure step width using a Kinect sensor. Their BA analysis revealed a bias of −0.015 m (left) and 0.027 m (right) and limits of ±0.076 m (left) and ±0.127 m (right). The present findings showed a slightly lower bias and similar limits compared to the left side of their results.

In the present study, the term swing width in relation to gait was introduced. The authors deemed it necessary to clearly separate step width and swing width, as both measures can deliver independent information about pathological gait. Step width delivers mainly information about the increase or decrease of the base of support. Whereas the swing width might be able to identify a circumduction [52], a common gait abnormality. Awada et al. [53] measured the circumduction of nine post-stroke patients using an OMC system and force plates and defined the severity of the gait impairment as the maximum lateral displacement of the center of gravity between stance and swing phase. As mentioned in Table 1, in the present study the swing width was defined as the minimal distance of the CA markers during the swing phase. Considering this assumption, it might appear in pathological gait that the anterior-posterior distance of the CA markers is smaller compared to the lateral distance, in the case of increased circumduction and decreased step length. Therefore, it might be beneficial to redefine the parameter swing width as the orthogonal distance between the line consisting of the CA marker of the standing foot and the line of progression, and the CA marker of the swinging foot.

However, there is little literature about the measurement of inter-foot distance during the swing phase of gait based on IMUs. Shiotani and Watanabe [54] examined a system using seven IMUs attached to the lower extremities based on Watanabe and Saito [55] for the measurement of circumduction during gait. They calculated the 3D loci vector of the thigh segments and graphically interpreted the circumduction behavior of 12 healthy subjects. However, they did not state quantitative results.

Bertuletti et al. [21] described an approach using a device consisting of an IMU and an infrared time of flight proximity sensor for the measurement of the step width. However, they actually measured the parameter that corresponds to the present definition of swing width. They validated the approach during the gait of one healthy subject. Their results were slightly better compared to the present findings of swing width, revealing a mean error of 0.005 m. However, the results of Bertuletti et al. [21] depend on a rather complex device including a large plastic plate target affixed to the opposite foot. Further, an optical calibration of the geometries of the sensor and its corresponding target is necessary.

### 4.2. Test-Retest Reliability

The calculation of the ICC revealed good to excellent test-retest reliability for all STP except for step width and swing width. Kluge et al. [18] and Donath et al. [25] found similar correlations in their calculated parameters. However, as mentioned afore, these authors described only a few STP.

The ICC values for step width and swing width were rated poor (0.25) and good (0.69), respectively. However, in the OMC system the test-retest reliability of these parameters was distinctively higher (0.67 and 0.90). Further, the step length revealed a lower ICC value (0.67) compared to the OMC system (0.88) and also the ICC value of the swing time was lower in the IMU system (0.73) compared to the OMC system (0.81). It seems not to be a coincidence that the STP that depend on the spatial information of both feet showed the lowest correlations concerning the test-retest reliability. It is likely that these parameters were most influenced by deviations in the neutral zero position calibration of Test 1 and the Retest, e.g., altered hip abduction or hip rotation. Robert-Lachaine et al. [56] examined the difference of self-placed and passively-placed neutral zero positions. They found an offset in the segment longitudinal axes for the lower limbs of about 5° to 10° between self-placement and passive-placement. In the present study, the neutral zero position was explained to the subjects but self-placed. There was no further passive correction of the poses, but verbal in exceptional cases. However, such deviations could not influence the measurements of the OMC system. This might explain the lower reliability concerning these STP in the IMU system.

## 5. Conclusions

In summary, the present study revealed valid results of an IMU system for 3D gait analysis delivering a wide range of clinically relevant parameters. Further, the real-time event-detection algorithm defined IC and TC with an error rate below 1.6% and an offset below 0.017 s. However, consider that in this study only young and healthy subjects participated. Therefore, the findings of this study apply only to normal gait. Future studies must show the validity and reliability of this approach in less standardized environments and examining subjects with gait impairment. Further, there is work in progress to introduce pose-independent and movement-independent calibration methods [45,46]. This could further improve the validity and reliability of STP like step width, swing width and step length. Additionally, the significance and the definition of acceptable resolutions concerning the step width and swing width have to be further discussed and examined in a clinical context.

## Figures and Tables

**Table 1:** Description of the spatio-temporal parameters (STP).

Parameter	Description
Step Length (m) *	Distance between the CA marker positions of the left and right foot projected on the ground at two consecutive contralateral ICs
Stride Length (m)	Distance between the CA marker positions of one foot projected on the ground at two consecutive ipsilateral ICs
Step Width (m) *	Orthogonal distance between the line of the CA marker positions of one foot projected on the ground at two consecutive ipsilateral ICs and the CA marker position of the contralateral foot at the contralateral IC
Swing Width (m) *	Minimal distance between both CA markers during the swing phase
Step Time (s)	Period between two consecutive ICs of the left and right foot
Stride Time (s)	Period between two consecutive ICs of the ipsilateral foot
Cadence (steps/min)	60 divided by step time
Single Limb Support (s)	Period between contralateral TC and contralateral IC
Double Limb Support (s)	Stride time minus Single limb support
Stance Time (s)	Period between IC and TC of one foot
Swing Time (s)	Period between TC and IC of one foot
Speed (m/s)	Stride length divided by Stride time [4]

An asterisk * marks the STP that require the spatial information of both feet for the calculation.

**Table 2:** Event-detection results for Test 1. The total of detected events, number of erroneous detections, false-positives and false negatives, percentage error and the offset between optical (OMC) and inertial measurement unit (IMU) system plus standard deviation (SD) are shown.

Test 1	Total	Total Errors	False-Positive	False-Negative	% Error	Offset (SD) (s)
IC	6619	1	1	0	0.02	0.008 (0.007)
TC	6796	80	44	36	1.17	0.016 (0.010)

**Table 3:** Summary of the results of all STP. The mean parameters plus SD, p-values, mean error, root mean square error (RMSE) (95% confidence interval (CI)), relative RMSE and bias (SD) are shown. Bold *p* values indicate significant differences between the systems (*p* < 0.05).

	OMC	IMU	*p* Value	Mean Error	RMSE	Relative RMSE (%)	Bias
Step Length (m)	0.61 ± 0.06	0.62 ± 0.07	**<0.05**	0.006	0.04 (0.03−0.04)	6.69	0.006 (0.08)
Stride Length (m)	1.21 ± 0.12	1.22 ± 0.12	0.39	0.005	0.04 (0.03−0.04)	2.98	0.005 (0.07)
Step Width (m)	0.09 ± 0.03	0.10 ± 0.03	**<0.05**	0.008	0.03 (0.02−0.03)	34.34	0.008 (0.06)
Swing Width (m)	0.09 ± 0.02	0.08 ± 0.03	**<0.05**	−0.008	0.03 (0.02−0.03)	35.20	−0.008 (0.06)
Step Time (s)	0.60 ± 0.06	0.60 ± 0.06	0.33	0.002	0.02 (0.01−0.02)	2.94	0.002 (0.03)
Stride Time (s)	1.20 ± 0.11	1.20 ± 0.11	0.63	0.002	0.01 (0.01−0.01)	0.90	0.002 (0.02)
Cadence (steps/min)	101.09 ± 10.02	100.79 ± 9.76	0.33	−0.296	3.10 (2.23−2.87)	3.07	−0.296 (6.05)
Single Limb Support (s)	0.39 ± 0.03	0.40 ± 0.03	**<0.05**	0.008	0.02 (0.01−0.02)	4.26	0.008 (0.03)
Double Limb Support (s)	0.81 ± 0.09	0.80 ± 0.09	**<0.05**	−0.006	0.02 (0.02−0.02)	2.32	−0.006 (0.04)
Stance Time (s)	0.80 ± 0.09	0.80 ± 0.09	**<0.05**	−0.008	0.02 (0.01−0.02)	2.10	−0.008 (0.03)
Swing Time (s)	0.39 ± 0.03	0.40 ± 0.03	**<0.05**	0.010	0.02 (0.01−0.02)	4.40	0.010 (0.03)
Speed (m/s)	1.03 ± 0.14	1.03 ± 0.15	0.57	0.003	0.03 (0.02−0.03)	2.72	0.003 (0.05)

**Table 4:** Event-detection results for the retest. The total of detected events, number of erroneous detections, false positives and false negatives, percentage error and the offset between OMC and IMU system plus SD are shown.

Retest	Total	Total Errors	False-Positive	False-Negative	% Error	Offset (SD) (s)
IC	6802	15	7	8	0.22	0.007 (0.008)
TC	6780	105	58	47	1.55	0.015 (0.010)

**Table 5:** Summary of the results of the calculation of the intraclass correlation coefficient for the OMC and IMU system, respectively.

Parameter	ICC OMC	ICC IMU
Step Length (m)	0.88	0.67
Stride Length (m)	0.87	0.88
Step Width (m)	0.67	0.25
Swing Width (m)	0.90	0.69
Step Time (s)	0.87	0.87
Stride Time (m)	0.92	0.91
Cadence (steps/min)	0.87	0.87
Single Limb Support (m)	0.82	0.85
Double Limb Support (m)	0.89	0.90
Stance Time (s)	0.92	0.92
Swing Time (s)	0.81	0.73
Speed (m/s)	0.91	0.92
